# Case Report: Influence of BRCA1 germline mutation on treatment-related morbidity of a non-seminomatous germ cell tumor patient

**DOI:** 10.3389/fonc.2025.1579574

**Published:** 2025-05-30

**Authors:** Bruno Griesler, Susann Schulze, Thomas Kegel, Christine Dierks, Pablo Villavicencio-Lorini, Markus Eszlinger, Haifa Kathrin Al-Ali, Nadja Jaekel

**Affiliations:** ^1^ University Clinic and Outpatient Clinic for Internal Medicine IV, University Medicine Halle (Saale), Halle (Saale), Germany; ^2^ Institute of Molecular Medicine, Martin Luther University Halle-Wittenberg, Halle (Saale), Germany; ^3^ Krukenberg Cancer Center Halle, Martin Luther University Halle-Wittenberg, University Medicine Halle, Halle (Saale), Germany; ^4^ Specialist Practice for Human Genetics, Medical Care Center (MVZ) University Hospital Halle gGmbH, Halle (Saale), Germany; ^5^ Institute of Clinical Genetics, Faculty of Health Sciences Brandenburg, Brandenburg Medical School Theodor Fontane, Neuruppin, Germany; ^6^ Institute of Pathology, University Medicine Halle (Saale), Halle (Saale), Germany

**Keywords:** hematotoxicity, chemotherapy - oncology, neutropenia and fever, case report, *BRCA1* mutation, GCT = germ cell tumor

## Abstract

We present the case of a 47-year-old male with advanced non-seminomatous germ cell tumor, who was found to carry a heterozygous pathogenic *BRCA1* germline variant following molecular testing due to a positive family history. While tumor analysis did not confirm loss of heterozygosity, evidence suggests that BRCA1 haploinsufficiency also increases genomic instability and cancer risk. After pre-phase treatment and the first cycle of chemotherapy, the patient developed prolonged pancytopenia leading to neutropenic sepsis. Subsequent cycles showed a shorter duration of pancytopenia, though it remained significant. A literature review indicates that BRCA1 deficiency may impair bone marrow recovery after chemotherapy, as observed in breast cancer patients, which we hypothesize also applies in this case. After first-line treatment, the patient had a partial response. In case of recurrence, the use of PARP inhibitors should be considered due to the BRCA1 deficiency.

## Introduction

With 2,001,140 new cancer cases and 611,720 cancer related deaths projected to occur in the United States in 2024, malignant diseases continue to be of outstanding importance for the healthcare system as well as for medical research. For germ cell tumors, with a comparatively good prognosis, around 10.000 new cases and 500 related deaths are projected for 2024 (1). Cancer treatment has developed rapidly in recent decades, leading to greater efficacy and tolerability by taking more and more cancer- and patient-specific characteristics into account (2).

*BRCA1* is one of the best-known tumor suppressor genes playing an essential role in the response to cellular stress by activating DNA repair processes (3). Mutations in the *BRCA1* gene are a common cause of hereditary ovarian and breast cancer (4). Other malignant diseases associated with pathogenic germline *BRCA1* variants include pancreatic and uterine cancer as well as Hodgkin’s disease. Prostate cancer, colorectal cancer and malignant melanoma are also frequently mentioned in connection to germline *BRCA1* mutations, but study results on these entities are contradictory or at least inconsistent between different age groups (5–9). The incidence of germ cell tumors (GCT) does not appear to be significantly increased in individuals with mutated *BRCA1* (10–12).

The gene product of *BRCA1* is a multifunctional protein, which is involved in DNA repair, cell differentiation, cell cycle checkpoint regulation, transcription regulation but also in DNA-independent processes like the regulation of glucose and lipid metabolism via the PI3K/AKT signaling pathway (13, 14). Given the numerous cellular processes in which *BRCA1* is involved, it seems plausible that a *BRCA1* mutation could influence not only the incidence of certain malignancies but also the course of therapy. In other malignant diseases, particularly breast, ovarian and pancreatic carcinomas, correlations between *BRCA1* mutation status and response to therapy, tendency to relapse and overall survival have been described (15, 16).

Patients with GCT have a very good prognosis compared to most other solid malignancies. However, in the case presented here, an unusually complicated course of first-line therapy was observed, which could be partly explained by an additional pathogenic *BRCA1* mutation.

## Case description

**Figure 1:**
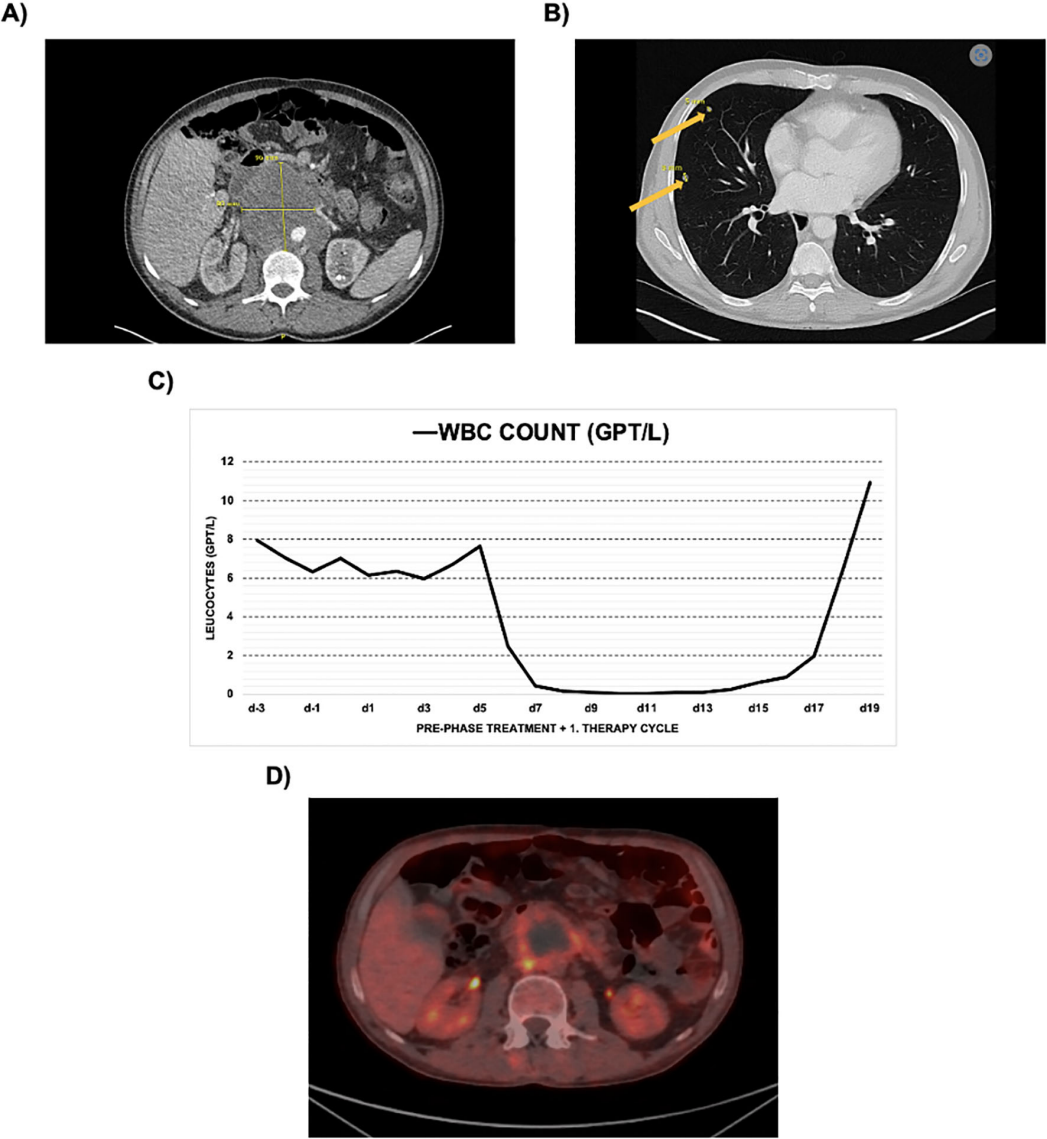
**(A)** CT scan of the upper abdomen showing the tumor bulk prior to treatment; **(B)** CT thorax showing exemplary marked pulmonary metastases; **(C)** Diagram showing the course of the white blood cell (WBC) count during pre-phase treatment (starting d-2) and the first therapy cycle (starting d1); **(D)** PET-CT scan after 4 therapy cycles showing a partial response.

A 47-year-old male was admitted to our hospital in November 2023 after an outpatient computer tomography (CT)-scan revealed a retroperitoneal mass (**Figure 1A**). Imaging was conducted due to persistent back pain and a 10% weight loss over the past two months. At time of admission, the patient was in stable general condition (Karnofsky Performance Status (KPS) 90%). The laboratory chemical analysis showed a highly elevated alpha-fetoprotein (AFP) plasma level (> 10,000 µg/l – normal range < 7 µg/l). A histological sample obtained via CT-guided biopsy confirmed a diagnosis of NSGCT. There was no clinical or sonographic evidence of testicular involvement, so the diagnosis of an extragonadal GCT was ultimately made. Further staging examinations revealed multiple pulmonary metastases (**Figure 1B**). Extrapulmonary metastases could not be confirmed. Due to the very high AFP level, the patient had to be classified in the high-risk group according to the *International Germ Cell Collaborative Group* prognostic classification system (IGCCCG) (17). At the uro-oncologic tumor board, it was decided to start a pre-phase treatment with carboplatin and etoposide to minimize the risk of a tumor lysis syndrome, followed by four cycles of PEB (cisplatin [d1-d5–20 mg/m^2^] + etoposide [d1-d5–100 mg/m^2^] + bleomycin [d1, d8, d15–30 mg]) chemotherapy.

As the patient had tested positive for the heterozygous pathogenic germline *BRCA1 frameshift*-variant NM_007294.4(BRCA1):c.843_846del (p.Ser282fs*15), resulting in a *loss-of-function* by nonsense-mediated decay, the case was also discussed in our molecular tumor board. Here it was recommended to start with PEB chemotherapy in curative intention and to consider the use of PARP inhibitors in case of refractory disease or relapse. The patient’s blood sample was tested for the above-mentioned BRCA1 germline mutation before admission to our clinic, as this had been detected in his mother, who had breast and ovarian cancer. Additionally, the histological sample of the NSGCT was tested positive for the germline BRCA1 mutation with a variant allele frequency (VAF) of 50.28% thus not confirming a loss of heterozygosity (LOH). Furthermore, a *KRAS* c.35G>T (p.G12V) mutation with a VAF of 42.82% was detected in the specimen, which fits well with a tumor cell content of 80%.

After a three-day pre-phase treatment (carboplatin [d1-3 - 100mg/m²] and etoposide [100mg/m²]) and the first cycle of PEB, the patient did not receive granulocyte colony-stimulating factor (G-CSF), as the risk of neutropenia is considered low with this regimen. However, severe leucopenia (with WBC < 1 Gpt/l to be assumed equal to neutropenia) was observed from day 8 of the first cycle of PEB, lasting for 8 days (see **Figure 1C**) (18). During this time, the patient developed fulminant sepsis on the basis of colitis with migratory peritonitis, which required intensive antibiotic therapy (vancomycin + meropenem + amikacin/tigecycline + ceftazidime) and temporary circulatory stabilization by volume substitution but no surgical intervention. Identification of the sepsis-causing bacteria was not successful. In the course of the disease, the patient developed a pronounced somnolence. There was no pathologic finding detectable by a CT-scan of the brain. Furthermore, there was no evidence of infectious meningitis or encephalitis, so we evaluated the somnolence in the context of a sepsis-associated encephalopathy. Other complications that occurred during the first cycle of therapy were hypokalemia requiring supplementation, nausea, and hypalbuminemia with peripheral edema. Due to the patient’s severely reduced general condition, bleomycin could not be administered on day 8 and day 15.

The complications that occurred during the first treatment cycle resulted in a hospitalization of approximately one month.

The following treatment cycles showed significantly less hematotoxicity with neutropenia of a maximum of 5 days. Septic constellations did not recur. However, pronounced hypokalemia and difficult-to-treat nausea were observed throughout the treatment period. G-CSF has not been used in any of the treatment cycles.

Regarding treatment response, the AFP value decreased to 32.7 µg/l after the second treatment cycle and to 8.5 µg/l after completion of first-line treatment. A positron emission tomography (PET)-CT after the fourth therapy cycle confirmed a partial response with PET-positivity remaining in the peripheral regions of the abdominal tumor bulk. The pulmonary metastases did not show signs of vitality post treatment (**Figure 1D**). In accordance with the guidelines, vital tumor remnants were surgically removed.

## Discussion

Pathogenic *BRCA1* germline variants can dramatically increase the risk of cancer development in affected individuals (19). The common theory of carcinogenesis in the case of *BRCA1* defects is based on the “two-hit” model (20). According to this model, if a *BRCA1* germline mutation is present, the “healthy” allele must first be compromised in the sense of LOH resulting in increased DNA double-strand breaks by homologous recombination deficiency (HRD) in order for a tumor to develop (21). However, this view is increasingly challenged by recent findings that even the haploinsufficiency of *BRCA1* and the resulting genomic instability are sufficient to convert healthy cells into malignant ones. According to this theory, the frequently observed LOH would be a consequence rather than a prerequisite for the development of *BRCA1*-haploinsufficient tumors (22, 23). The *BRCA1* gene has also been found to be frequently methylated in GCT (24, 25). Against the background of these findings, it is likely that even if GCT, not belonging to the classic “BRCA1 cancer entities”, the existing germline mutation in our patient contributed to the development and progression of the NSGCT.

Systemic *BRCA1* haploinsufficiency in individuals with respective germline mutations also appears to influence the frequency and severity of chemotherapy side effects.

In this context, hematotoxicity should be mentioned in particular. Hematopoietic stem cells are highly dependent on continuous DNA repair, which is realized to a relevant extent by *BRCA1* (26). Biallelic loss-of-function mutations of *BRCA1* result in Fanconi anemia, complementation group S (27). However, Mgbemena et al. showed that in a mouse model, the loss of one functional *BRCA1* allele leads to a significantly lower blood count and a reduced regenerative capacity of the bone marrow (28). Breast cancer patients with a heterozygous pathogenic *BRCA1* germline variant appear to have significantly higher hematotoxicity than patients without the corresponding mutation. However, this does not seem to apply to ovarian cancer patients. The authors of the mentioned study explain this by the fact that ovarian cancer patients were often treated with platinum derivative monotherapy, while breast cancer patients usually received at least two DNA-damaging therapeutic agents (29). In breast cancer patients, the development of agranulocytosis and febrile neutropenia after the first chemotherapy cycle has even been shown to be an independent predictive factor for the detection of a BRCA1 germline mutation (30).

The treatment regimen according to which our patient was treated (CE pre-phase + PEB) consisted exclusively of DNA-damaging chemotherapeutic agents. However, since the risk of febrile neutropenia (FN) in the treatment of GCT with PEB is less than 20%, routine FN prophylaxis is not recommended (31). The patient’s *BRCA1* germline variant may have been a factor influencing hematotoxicity under chemotherapy. However, it should be noted that the patient with his age of over 35 years and an initial tumor diameter of > 60 mm, also meets additional factors associated with an increased risk of FN under GCT treatment (32).

Reduced expression of functional BRCA1 could also influence the response to therapy with DNA-damaging substances (33–36). However, the tumor type seems to play a role here. For example, while *BRCA1*-deficient ovarian carcinomas and triple-negative breast carcinomas show a good response to therapy, this appears to be the opposite for *BRCA1*-deficient non-triple-negative breast carcinomas and lung carcinomas (37). To our knowledge, there is no data on how *BRCA1*-deficiency modulates the therapy response of NSGCT. However, the majority of GCT is intrinsically hypersensitive to cisplatin due to impaired DNA repair and highly active pro-apoptotic pathways. In up to 15% of cases, platinum resistance develops over the course of the disease, which is caused by various mechanisms, including upregulated DNA repair (38). Members of the PARP family, which are frequently overexpressed in GCT, are involved in DNA maintenance by base excision repair (39). The use of PARP inhibitors in therapy-refractory germ cell tumors was investigated in two clinical phase II studies. Both, monotherapy with the PARP inhibitor olaparib and combination therapy with the PARP inhibitor veliparib, gemcitabine and carboplatin showed only minor therapeutic effects (40, 41).

PARP inhibitors have already been successfully used in *BRCA1*-deficient tumors such as pancreatic, prostate, breast and ovarian carcinomas. The postulated molecular mechanism here is that DNA repair, which is already compromised by the loss of BRCA1, is further reduced by inhibiting the partially compensatory PARP proteins. Then, the resulting accumulation of DNA damage leads to significantly increased cell death (41).

In the event of recurrence after potential second-line treatment and auto-transplant, the use of PARP inhibitors could be a promising approach in the patient described. Although the data available today for the use of PARP inhibitors in advanced germ cell tumors are rather unpromising, these drugs could show a relevant therapeutic effect against the background of the existing *BRCA1* variant. With regard to the somatic *KRAS* G12V mutation found in the tumor, it has to be stated there is currently no specific inhibitor for this variant in clinical use. However, Pan-KRAS inhibitors have been developed recently and are being tested pre-clinically right now (42). Furthermore, pharmacologic targeting of MEK as a down-stream target of KRAS has been shown to be effective in multiple *KRAS*-mutated malignant tumors (43–45). This could be another therapeutic option to be discussed in case of relapse.

## Conclusion

*BRCA1* germline variants not only alter the risk of developing certain types of cancer, but also affect the course of cancer treatment. *BRCA1* haploinsufficiency, which is also present in bone marrow stem cells, could lead to delayed hematologic regeneration after the use of DNA-damaging substances. As this can also increase the risk of febrile neutropenia, the use of G-CSF prophylaxis should be given particular consideration (29). Pre-phase treatments, as used in the case described above, could reduce the risk of tumor lysis syndrome, but also increase the hematotoxicity. Therefore, pre-phase treatments should be critically discussed in patients with *BRCA1*-deficiency.

However, the germline *BRCA1* mutation as well as the somatic *KRAS* G12V mutation represent quasi-targetable genetic alterations that might justify the use of PARP or MEK inhibitors in the case of a relapse.

This case report could be understood as an indication that *BRCA1* mutation is not only important in the “classic BRCA1 entities” but also represents a general influencing factor in cancer therapy. Of course, it is in the nature of a case report that only indications, but no statistically reliable findings can be derived from it. Both preclinical and clinical studies are needed to further determine the relevance of BRCA1 haploinsufficiency in the context of chemotherapy-induced hemotoxicity.

## Data Availability

The raw data supporting the conclusions of this article will be made available by the authors, without undue reservation.
